# Molecular cytogenetic identification and phenotypic description of a new synthetic amphiploid, *Triticum timococcum* (A^t^A^t^GGA^m^A^m^)

**DOI:** 10.1007/s10722-014-0135-0

**Published:** 2014-06-19

**Authors:** Péter Mikó, Mária Megyeri, András Farkas, István Molnár, Márta Molnár-Láng

**Affiliations:** Agricultural Institute, Centre for Agricultural Research, Hungarian Academy of Sciences, PO Box 19, 2462 Martonvásár, Hungary

**Keywords:** FISH karyotype, Multicolour GISH, Synthetic amphiploid, *Triticum monococcum*, *Triticum timococcum*, *Triticum timopheevii*

## Abstract

A recently developed synthetic amphiploid, *Triticum timococcum* Kost., nom. nud. (2n = 6x = 42, A^t^A^t^GGA^m^A^m^) is described in the present study. This hexaploid taxon was developed by colchicine treatment in Martonvásár from the hybrid of a selected accession of *Triticum timopheevii* Zhuk. (2n = 4x = 28, A^t^A^t^GG) and a prebred semi-dwarf line of *Triticum monococcum* L. (2n = 2x = 14, A^m^A^m^). A detailed cytomolecular examination was carried out using the sequential multicolour fluorescence and genomic *in situ* hybridization techniques (FISH and mcGISH). It was proved that *T. timococcum* has 42 chromosomes originating from its parents. The chromosomes of the A genomes of *T. monococcum* and *T. timopheevii* could be distinguished in the amphiploid using FISH. The successful discrimination of the chromosomes was supported by the karyotypes of the three genomes and the successful optimization of the mcGISH technique for the A and G chromosomes achieved in the present study. A phenotypic evaluation was also carried out under natural and artificial growing conditions in 2012 and 2013. Based on the results, *T. timococcum* has intermediate characteristics in terms of spike (spikelet) shape and plant height, while it is similar to the female parent, *T. timopheevii* regarding pubescence. Like its parents, *T. timococcum* showed outstanding resistance to the main fungal diseases of wheat. *T. timococcum* headed later and developed longer and looser spikes, fewer tillers and only a third as many seeds than its parents. The third generation of *T. timococcum* was able to develop an acceptable number of seeds, even taking into account the reduced germination ability in the field.

## Introduction

As the effects of global climate change escalate, prebreeding becomes increasingly important in the improvement of bread wheat (*Triticum aestivum* L. subsp. *aestivum*). Wild relatives of wheat carry many useful resistance genes against biotic and abiotic stresses that could be incorporated into the wheat genome in order to make wheat cultivation more secure and to reduce the negative impact of intensive cultivation on the natural environment (Arraiano et al. 2001; Pestsova et al. 2001; Mujeeb-Kazi and Rajaram 2002). It is well known that one possible way of increasing the genetic diversity of prebreeding materials is the development of synthetic amphiploid wheat lines. These lines are interspecific hybrids that have a diploid set of chromosomes originating from each parental species. Besides direct crosses between bread wheat and one of its wild relatives, an increase in the genetic diversity can also be achieved by developing synthetic amphiploid wheat lines from two wild relatives (Sears 1981). One such amphiploid is synthetic hexaploid wheat, arising from the re-synthesis of bread wheat (Mujeeb-Kazi et al. 1996; Zhang et al. 2005; Lage et al. 2006; Jones et al. 2013).

The combination of *Triticum timopheevii* Zhuk. and *Triticum monococcum* L. subsp. *monococcum* (einkorn wheat) could be an excellent way to develop new synthetic amphiploids for the establishment of a promising prebreeding approach.

The tetraploid (2n = 4x = 28, A^t^A^t^GG) *T. timopheevii* is a well-known source of resistance genes (Mujeeb-Kazi and Kimber 1985; Belea 1992), making it resistant to fungal diseases such as powdery mildew (*Blumeria graminis* (DC.) Speer), leaf rust (*Puccinia triticina* Erikss.), stem rust (*Puccinia graminis* Pers.) and smut [*Ustilago tritici* (Pers.) Rostrup] (McIntosh and Gyárfás 1971; Järve et al. 2002). This species also has outstanding tolerance to abiotic stresses, such as drought. Moreover, the cultivated forms of this species have good bread-making quality with high protein content (Zhukovsky 1971).

The diploid (2n = 2x = 14, A^m^A^m^) einkorn wheat also carries effective resistance genes against most of the main fungal diseases of wheat (e.g. powdery mildew, leaf rust) (Vavilov 1949; The and Baker 1975; Monneveux et al. 2000). Most cultivated einkorn lines have good winter hardiness and allelopathic effect, and also tolerate drought well (Megyeri et al. 2011). The valuable components (carotenoids, tocol) in the grains make einkorn wheat a promising source for healthy food production (Brandolini et al. 2008).

An earlier detailed phenotypic description combined with the prebreeding approach have resulted in the selection of the most promising of the 56 *T. timopheevii* accessions preserved in the Martonvásár Cereal Gene Bank. As a further result of this approach, a *T. timopheevii* × *T. monococcum* hybrid has been developed between this accession and a previously prebred semi-dwarf einkorn line (Mikó et al. 2013). Following the suggestion of Goncharov et al. (2009), this amphiploid is known as *Triticum timococcum* Kost., nom. nud.

The present study is focusing on the molecular cytogenetic identification of the genome composition and on the examination and phenotypic description of this new synthetic amphiploid, *T. timococcum*.

## Materials and methods

### Plant material

The female parent was *T. timopheevii* Zhuk. subsp. *timopheevii* var. *rubiginosum* (Accession No.: MVGB845), while the male parent was a semi-dwarf einkorn line (*T. monococcum* L. subsp. *monococcum* ‘1T-1’) having good agronomic characteristics. This einkorn line was bred in Martonvásár, and has relatively good crossability with other *Triticum* species (Kovács et al. 2012; Megyeri et al. 2011). A hybrid between these selected plant materials was developed in recent years in Martonvásár and named *T. timococcum* Kost., nom. nud. The fertile hexaploid genome of the new hybrid was doubled by colchicine treatment, so the further generations are referred to as C_n_, instead of F_n_. Several plants of the *T. timococcum* C_2_ (progenies of treated F_1_ generation) and C_3_ generations were studied. Disease resistance studies were carried out using sensitive cultivars of bread wheat as controls in the field (Mv Emese) and in the phytotron (Alcedo).

Two gene bank accessions were also sown in the nursery in Martonvásár so that a sufficient amount of total genomic DNA could be isolated for the molecular cytogenetic analysis. The donors of the A and S genomes were *Triticum urartu* Tumanian ex Gandilyan (Acc. No.: MVGB115) and *Aegilops speltoides* Tausch var. *speltoides* (Acc. No.: MVGB905), respectively. The botanical names used in the present study are based on the wheat classification system of van Slageren (1994).

### Fluorescence *in situ* hybridization

Mitotic metaphase chromosome preparations were made from the root tips of germinating seeds of the doubled hybrid (hexaploid) progenies as previously described by Jiang et al. (1994), while fluorescence *in situ* hybridization (multicolour FISH and GISH) was carried out according to Molnár et al. (2009). In the case of FISH the repetitive DNA sequences pSc119.2 (Bedbrook et al. 1980) and Afa-family (Nagaki et al. 1995) were labelled with biotin-16-dUTP (Roche Diagnostics GmbH, Mannheim, Germany) and digoxigenin-11-dUTP (Roche), respectively, and they were amplified by PCR (Nagaki et al. 1995; Contento et al. 2005). The 18S-5.8S-26S rDNA clone pTa71 (Gerlach and Bedbrook 1979) was labelled with biotin-16-dUTP and digoxigenin-11-dUTP in a ratio of 1:1. FISH was followed by multicolour genomic *in situ* hybridization (mcGISH). After documenting the FISH patterns of entire cells, the slides were washed and rehybridized using the *T. urartu* and *Ae. speltoides* genomic probes. Total genomic DNA was extracted from the plants using the phenol–chloroform method described by Sharp et al. (1988). During the development of the hybridization probes, the genomes of *Ae. speltoides* (genome S, ancestor of genome G) and *T. urartu* (genome A) were labelled by nick translation with digoxigenin-11-dUTP and biotin-16-dUTP, respectively. Before hybridization, all the total genomic DNAs were sheared by autoclaving for 4.5 min. For FISH 0.4 μl Afa-family, 0.6 μl pSc119.2 and 0.6 μl pTa71 probes were added to the hybridization mixture of each slide, while the concentration and ratio of the total genomic probes in the hybridization mixture of mcGISH were optimized in a preliminary study, on the basis of which 40 ng (0.8 μl) labelled A genomic DNA probe, 40 ng (0.8 μl) labelled S genomic DNA probe and unlabelled blocking DNA from S genomic DNA at 50 times the quantity of the probes (2 μg) were added to the hybridization mixture of each slide. The concentration of the S genomic DNA used as blocking DNA was 2,793 ng/μl, so 0.716 μl/slide was added to the hybridization solution.

After the *in situ* hybridizations (FISH or mcGISH), the detection of digoxigenin and biotin was carried out using anti-digoxigenin-rhodamine Fab fragments (Roche) and streptavidin-FITC (Roche), respectively (Molnár-Láng et al. 2010). The labelled chromosomes were examined with a Zeiss AxioImager.M2 fluorescence microscope using a Plan Neofluar oil objective × 100, NA 1.3 (Carl Zeiss Microimaging GmbH, Göttingen, Germany) equipped with filter sets appropriate for DAPI (Zeiss filter set 49), FITC (Zeiss filter set 38) and rhodamine (Zeiss filter set 20). The images were captured with a Zeiss AxioCam MRm CCD camera and compiled with AxioVision 4.8.2 software.

The discrimination of A genome chromosomes was based on the findings of Megyeri et al. (2012), while the identification of G chromosomes was aided by the results of Uhrin et al. (2012).

### Phenotypic description; statistical analysis

Field assessment was carried out in the certified organic nursery of the gene bank in Martonvásár in 2012 and 2013. Twenty seeds were sown in each 1-m row in October (row distance: 20 cm). Precipitation in the field during the vegetation season (15th October to 15th July) amounted to 238 and 371 mm, respectively, in 2012 and 2013. In parallel, plants of the parents and their synthetic hybrids were grown in pots in a phytotron chamber (Conviron PGR-15 cabinet) under controlled environmental conditions, where the growth parameters were adjusted according to Molnár-Láng et al. (2012). Seedlings germinated in jiffy pots were put in the climate chamber after 6 weeks’ vernalization at 4 °C in pots measuring 18 cm in height and 11 cm in diameter.

In 2012, seeds from the best 53 isolated spikes of *T. timococcum* were sown without seed treatment in distinct rows in the field next to the rows of the parents. In parallel, 31 seeds from 7 of the 53 *T. timococcum* spikes were grown in the phytotron together with 3–4 plants of their parents. The phenotypic description of the plant material was continued in 2013 by assessing 56 rows of *T. timococcum* (C_3_ generation) together with 10 plants of each parent, and 31 *T. timococcum* plants in a phytotron chamber without the parents. All the spikes in the phytotron and the main spikes of 30 randomly selected plants from each genotype were also analysed (except *T. timococcum* in the field in 2012).

Growth habit (winter, spring or facultative type) and heading date (only in the field) were examined during the vegetation season, while the maximum plant height (in cm from the ground to the top of the awns) and number of tillers (spikes) per plant were determined immediately before harvest. Spike length (without awns), spikelet density (number of spikelets per 1 cm of spike), number of florets per spike and number of seeds per spike were measured after harvest. In both years, spikes of *T. timococcum* were isolated (except in the field in 2013), while spikes of the parents were not.

The morphological traits of *T. timococcum* were compared with those of the parents using *t* test to detect the significance of the difference between the means of two independent samples. Significant differences between the two consecutive generations (years) of *T. timococcum* were also analysed.

### Disease resistance studies

Resistance to frequently occurring wheat diseases (powdery mildew, leaf rust and yellow rust) was examined in 2013, when a sensitive bread wheat cultivar (*T. aestivum* subsp. *aestivum* ‘Mv Emese’) was used as a control in the field. In addition, artificial leaf rust inoculation was carried out on 3-leaf plantlets in the phytotron during the winter of 2012/2013 using a sensitive bread wheat cultivar, Alcedo as a control. Leaf rust infection was scored according to Stakman et al. (1962).

## Results

### Genome composition

#### FISH karyotype

**Fig. 1:**
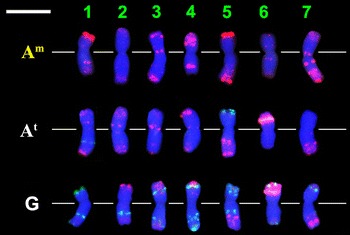
FISH Karyotypes of A^m^, A^t^ and G genomes: fluorescence *in situ* hybridization patterns of repetitive DNA probes pSc 119.2, Afa family and pTa71 on chromosomes of *Triticum monococcum* L. subsp. *monococcum* 1T-1 (A^m^) and *T. timopheevii* Zhuk. subsp. *timopheevii* var. *rubiginosum* MVGB845 (A^t^ and G) arranged according to genomes and homeologous groups (*bar* = 10 μm)

Three-colour FISH was carried out separately on the female parent, *T. timopheevii* (2n = 4x = 28, A^t^A^t^GG) and on the male parent, *T. monococcum* (2n = 2x = 14, A^m^A^m^), in order to obtain appropriate “raw material” for developing an effective karyotype for the A^t^, G and A^m^ genomes. Repetitive DNA probes pSc119.2, Afa family and pTa71 were used simultaneously in the hybridization, and the labelled chromosomes of the somatic metaphase cells were photographed. The individual chromosomes were then cut out and assembled to show the karyotypes of the three genomes used for the identification of *T. timococcum* chromosomes (Fig. 1).

Chromosome discrimination was carried out using the previously published FISH patterns of *T. timopheevii* (Jiang and Gill 1994; Uhrin et al. 2012) and *T. monococcum* (Megyeri et al. 2012). Chromosomes 6G, 6A^t^, 1A^m^ and 5A^m^ showed very strong pTa71 signals, while the green coloured pSc119.2 probe gave strong signals on all the G genome chromosomes and weaker signals on two A^t^ chromosomes (1A^t^ and 5A^t^). In combination with the Afa-family signals, this allowed all the G genome chromosomes to be clearly distinguished from each other (Fig. 1). The Afa-family probe hybridized mainly to the chromosomes of the A^m^ and A^t^ genomes, which could be distinguished from each other using these karyotypes and the detailed description created from them (Table 1).

**Table 1:** Hybridization sites of the digoxigenated Afa-family (and other) repetitive DNA sequences on the different A genomes of *T. timococcum* Kost., nom. nud

Chromo-some	Afa-family (and other FISH) signals of the A^m^ chromosome originating from *Triticum monococcum* L. subsp. *monococcum* 1T-1		Afa-family (and other FISH) signals of the A^t^ chromosome originating from *Triticum timopheevii* Zhuk. subsp. *timopheevii* var. *rubiginosum* MVGB845	
	Short arm (and centromere)	Long arm	Short arm (and centromere)	Long arm
1	Some signals in the terminal region; (strong pTa71 signal at the terminal end)	Two signals in the interstitial region; weak signals in the terminal region	Some weak signals in the terminal region	Two signals at the terminal end; (a pSc119.2 band in the interstitial region)
2	Very weak signals at the terminal end	Very weak signals at the terminal end	Two signals near the centromere; weak signals in the terminal region	Signals in the terminal region
3	Two signals in the terminal region; Two signals near the centromere	A few signals at the terminal end	Two signals near the centromere	Some weak signals in the terminal region
4	Many strong signals	Two subterminal signals	Two signals at the terminal end; Two weak signals in the centromere	Weak signals in the terminal region
5	(Strong pTa71 signal in the terminal end)	Two strong signals at the terminal end; weak signals in the terminal region	(Two strong pSc119.2 signals at the terminal end)	Two signals in the terminal region; Two signals in the interstitial region; weak signals between them
6	Weak signals at the terminal end; weak signals near the centromere	Weak signals at the terminal end; weak signals near the centromere	Some signals at the terminal end; (strong pTa71 band in the subterminal region)	No signals
7	Weak signals at the terminal end; Two signals in the centromere	Many signals in the terminal region; Two signals near the centromere	No signals	Signals in the terminal region; Two weak signals near the centromere

#### Genome of *Triticum timococcum*

**Fig. 2:**
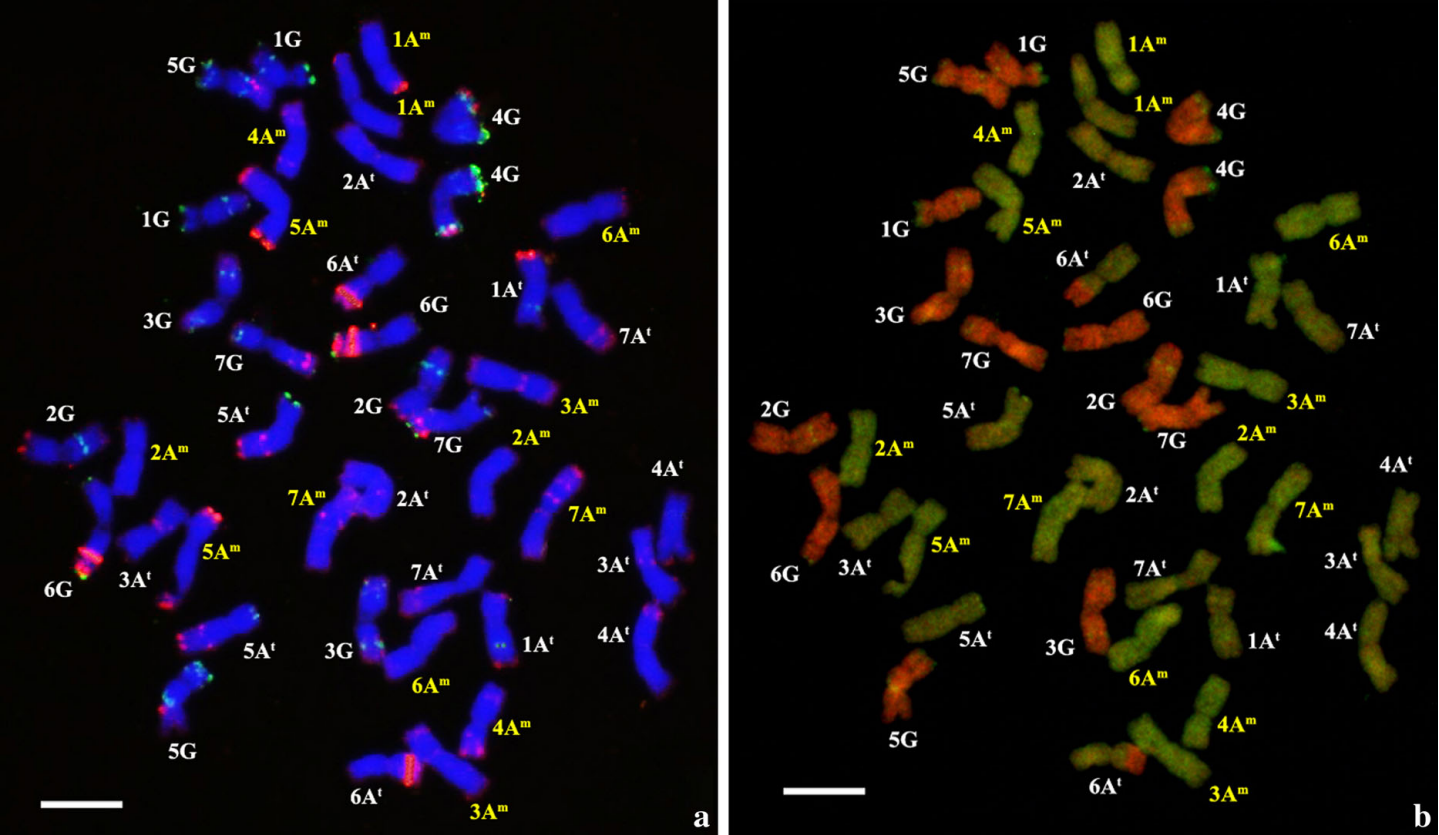
FISH (**a**) and mcGISH (**b**) patterns on mitotic chromosomes of the same hexaploid *T. timococcum* Kost., nom. nud. C_2_ cell using labelled repetitive (**a**) and total genomic (**b**) DNA probes. Chromosomes labelled with white are from the *T. timopheevii* Zhuk. subsp. *timopheevii* var. *rubiginosum* MVGB845 parent, and chromosomes labelled with yellow are from the *Triticum monococcum* L. subsp. *monococcum* 1T-1 parent (*bar* = 10 μm)

Pretrials on *T. timopheevii* preparations proved the effectiveness of the combined FISH and mcGISH techniques for the discrimination of the A and G chromosomes. Chromosome counting was also carried out on metaphase spreads before the *in situ* hybridization, and most of the hybrid *T. timococcum* plants examined were found to have a stable hexaploid genome. The identification of the genome of the synthetic amphiploid, using the same FISH technique as for its parents, proved that normal doubling occurred after colchicine treatment, resulting in 42 chromosomes in the C_2_ generation. The whole sets of *T. timopheevii* and einkorn chromosomes could be clearly discriminated using the previously developed karyotypes (Fig. 2a). Based on the karyotypes and Table 1, all the seven A^m^ and seven A^t^ chromosomes could be identified in the amphiploid using FISH, mainly with the help of the Afa-family probe. Only chromosomes 2A^m^ and 6A^m^ were difficult to distinguish, though 6A^m^ exhibited slightly more of the very weak red-labelled Afa-family signals than 2A^m^. The green-labelled pSc119.2 probe hybridized mostly to G genome chromosomes, giving distinctive patterns on each of them. In addition, this probe also gave signals on chromosomes 1A^t^ (interstitial band on the long arm) and 5A^t^ (2 signals at the terminal end of the short arm). The hybridization patterns of pTa71 were found at the terminal end of the short arms of 1A^m^ and 5A^m^, and on the subterminal region of the short arms of 6A^t^ and 6G.

After stringency washing of the FISH slide, the genome composition of the *T. timococcum* plants was also analysed by mcGISH using the results obtained from the mcGISH optimization pre-study of *T. timopheevii*. The optimal hybridization mixture allowed clearly detectable signals to be obtained with high contrast. During this procedure the green-coloured *T. urartu* DNA and the red-coloured *Ae. speltoides* DNA hybridized to the A (A^t^ and A^m^) and G genomes, respectively, so both A genomes could be clearly discriminated from the G genome (Fig. 2b). Like its female parent, *T. timococcum* carries the same species-specific translocations previously reported by Rodríguez et al. (2000) and Uhrin et al. (2012). The short arm of chromosome 6A^t^ has a long translocated segment from the short arm of chromosome 1G, while 1G has a short segment from 6A^t^ (6A^t^S/1GS). McGISH also revealed that 4GS has a tiny segment from the A^t^ genome, which probably originated from the long arm of chromosome 4A^t^ (4GS/4A^t^L), as demonstrated by Rodríguez et al. (2000).

On the basis of these results, the genome composition of the newly developed synthetic amphiploid, *T. timococcum* can be described as 2n = 6x = 42, A^t^A^t^GGA^m^A^m^.

### Phenotypic traits

The results of the morphological description can be seen in Table 2 (field) and Table 3 (phytotron).

**Table 2:** Morphological traits of *T. timococcum* Kost., nom. nud. grown in the field compared to its parents and to its previous generation (2012 and 2013, Martonvásár)

Plant material assessed	Year	Number of plants/spikes evaluated	Heading date	Plant height (cm)	Tillering (spikes/plant)	Length of spike (cm)	Florets/spike	Seeds/spike	Density of spikelets (spikelets/cm of spike)
*Triticum timopheevii* Zhuk. subsp. *timopheevii* var. *rubiginosum* MVGB845	2012	10/30	28 May	109.3	9.4	5.48	42.0	29.7	3.84
	2013	10/30	13 June	96.9	12.4	6.47	45.3	33.4	3.52
*Triticum monococcum* L. subsp. *monococcum* 1T-1	2012	10/30	24 May	64.7	11.9	4.85	22.8	8.5	4.71
	2013	10/30	21 May	67.5	7.0	6.83	30.6	47.7^¤^	4.50
*T. timococcum*	2012	294/146	11 June**^,##^ (29 May-30 June)	71.9**^,××^	4.8**^,##^	6.40**^,##,××^	33.2**^,##,××^	13.0**^,##,×^	2.61**^,##,×^
	2013	458/30	14 June^##^	82.1*^,#,××^	4.5**^,#^	8.40**^,##,××^	41.7*^,##,××^	9.7**^,##,×^	2.49**^,##,×^

* Significantly different from MVGB845 in the same year at the *P* = 0.05 level

** Significantly different from MVGB845 in the same year at the *P* = 0.01 level

^#^Significantly different from 1T-1 in the same year at the *P* = 0.05 level

^##^Significantly different from 1T-1 in the same year at the *P* = 0.01 level

^×^Significantly different from *T. timococcum* in the other year at the P = 0.05 level

^××^Significantly different from *T. timococcum* in the other year at the *P* = 0.01 level

^¤^Under good conditions the semi-dwarf line of einkorn develops not 1, but 2 florets (seeds) in 30–50 % of its spikelets

**Table 3:** Morphological traits of *T. timococcum* Kost., nom. nud. grown in the phytotron compared to its parents and to its previous generation (2012 and 2013, Martonvásár)

Plant material assessed	Year	Number of plants/spikes evaluated	Plant height (cm)	Tillering (spikes/plant)	Length of spike (cm)	Florets/spike	Seeds/spike	Density of spikelets (spikelets/cm of spike)
*Triticum timopheevii* Zhuk. subsp. *timopheevii* var. *rubiginosum* MVGB845	2012	3/14	114.5	5.7	4.69	38.6	25.1	4.13
*Triticum monococcum* L. subsp. *monococcum* 1T-1	2012	4/20	70.6	5.0	6.25	33.4	25.1	5.43
*T. timococcum*	2012	31/88	119.7^##,××^	4.9^××^	6.67**^,×^	42.0^##^	10.6**^,##^	3.16**^,##,×^
	2013	31/67	82.7^××^	3.1^××^	6.22^×^	41.7	8.7	3.35^×^

* Significantly different from MVGB845 in 2012 at the *P* = 0.05 level

** Significantly different from MVGB845 in 2012 at the *P* = 0.01 level

^#^Significantly different from 1T-1 in 2012 at the *P* = 0.05 level

^##^Significantly different from 1T-1 in 2012 at the *P* = 0.01 level

^×^Significantly different from *T. timococcum* in the other year at the *P* = 0.05 level

^××^Significantly different from *T. timococcum* in the other year at the *P* = 0.01 level

In the 2011/2012 season 50.5 % of the *T. timococcum* seeds germinated in the field after sowing, and 80.8 % of them showed very good winter hardiness. The heading of *T. timococcum* lasted for a month in both years, the average heading date being 238 days after sowing. Based on the observations, the heading date of *T. timococcum* is less sensitive to the year effect than that of the female parent, and seems to be more like *T. monococcum*.

Strong significant differences were found for most of the traits examined in the field for 2 years. In 2012, only the plant height of the *T. timococcum* C_2_ generation did not differ significantly (*P* > 0.05) from that of the male parent. However, in 2013 *T. timococcum* showed a significant difference at the *P* = 0.05 level from both its parents for plant height, which can be explained by the 55 % greater precipitation in that year. Moreover, the two *T. timococcum* generations examined in the 2 years in both environments also showed significant differences (*P* < 0.05) for most of the traits, which could indicate the morphological instability of the early generations of this new synthetic amphiploid.

**Fig. 3:**
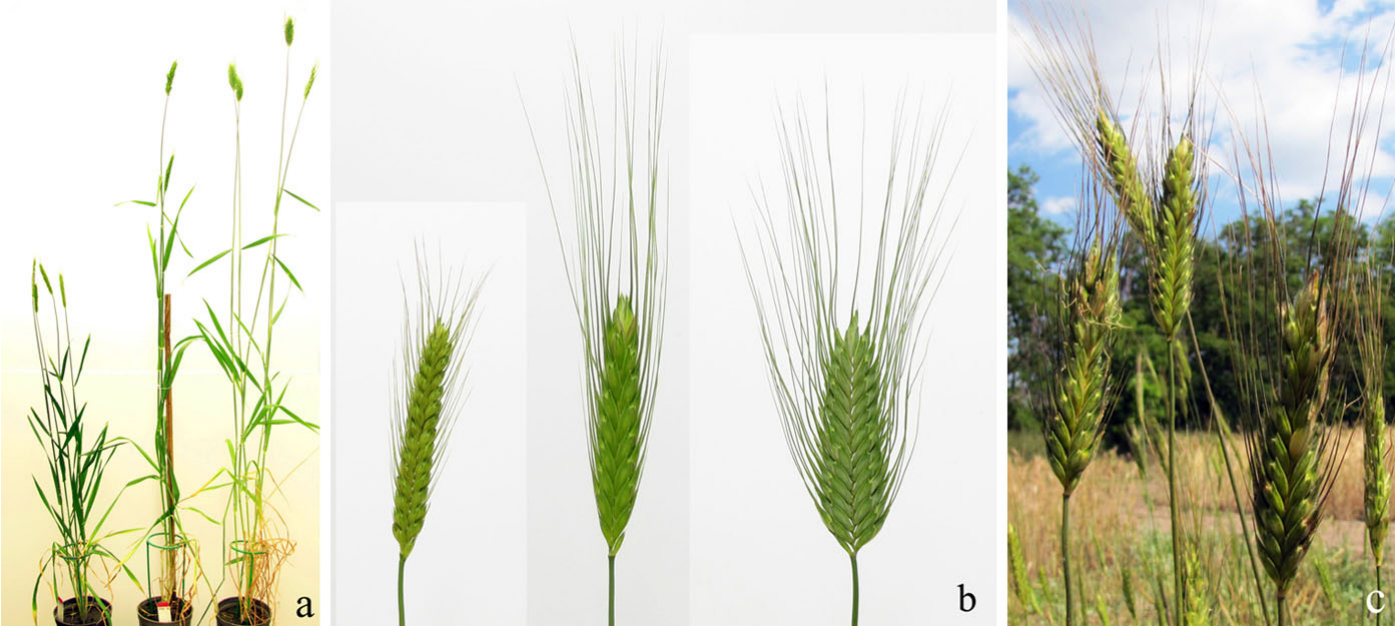
Whole plants of *Triticum monococcum* L. subsp. *monococcum* 1T-1 (*left*), *T. timococcum* Kost., nom. nud. (*middle*) and *T. timopheevii* Zhuk. subsp. *timopheevii* var. *rubiginosum* MVGB845 (*right*) grown in a climate chamber (**a**). Spikes of *T. monococcum* 1T-1 (*left*), *T. timococcum* (*middle*) and *T. timopheevii* MVGB845 (*right*) collected from the field (**b**). Double-tipped spike (*front left*) and slightly twisted spike (*front right*) of *T. timococcum* C_3_ plants in the field (**c**), Martonvásár, Hungary, 2013

The comparison of *T. timococcum* with its parents in the phytotron (only in 2012) showed significant differences for fewer traits. Unlike the field studies, the plant height of *T. timococcum* showed a greater resemblance to that of *T. timopheevii* in the phytotron (Fig. 3a).

In terms of spikelet density, *T. timococcum* developed longer and looser spikes than its parents. As the female parent of *T. timococcum* was a *T. timopheevii* accession, the inherited cytoplasmic (partial) male sterility of the hybrid resulted in a relatively low (around 30 %) average seed set. Moreover, half the seeds were unable to germinate in the field, while the germination rate in jiffy pots was almost 100 %. This could indicate the necessity for seed treatment in future field experiments on *T. timococcum*.

The growth habit of *T. timococcum* was also examined by assessing spring-sown plants in 2013, and it was found to be of the facultative type, like its parents.

*T. timococcum* was found to be closely similar to *T. timopheevii* regarding pubescence, because the whole plant was densely pubescent, which is one of the main characteristics of the species. Moreover, *T. timococcum* had more and thicker hairs than its female parent.

Regarding spike morphology, *T. timococcum* showed an intermediate type in comparison to its parents: the length and width of the spikelets were intermediate to that of the parents. *T. timococcum* had long awns and a flattened spike, like *T. timopheevii*, but the width of the spikes was more similar to that of einkorn (Fig. 3b).

In addition, some of the spikes were slightly twisted spirally along the longitudinal axis, and 1–2 % of the *T. timococcum* spikes were found to have a double tip (Fig. 3c).

### Disease resistance

As no leaf diseases could be observed in the organic nursery in 2012 (weather conditions not conducive to fungi), artificial leaf rust inoculation was carried out on the third generation of *T. timococcum* and its parents using plantlets grown in jiffy pots. This winter trial proved the high resistance of *T. timococcum* and its parents to leaf rust (scores of 0 or ;), compared to the sensitive bread wheat cultivar, Alcedo (score of 4, the maximum). This result was confirmed under field conditions in 2013, where the sensitive wheat cultivar Mv Emese had the maximum score of 4, while the neighbouring *T. timococcum* C_3_ plants and their parents showed no susceptibility to leaf rust. Moreover, no other major wheat leaf diseases (yellow rust, powdery mildew) could be observed on them during the vegetation period in 2013, while Mv Emese exhibited susceptibility to these diseases, too.

## Discussion

Several studies have dealt with synthetic amphiploids carrying the G genome and having unusual genome constitutions (e.g. Badaeva et al. 1995; Cao et al. 2000; Goncharov 2002; Laikova et al. 2004; Belea et al. 2005; Goncharov et al. 2007). Some of these authors examined the hexaploid *T. timococcum*, which was first developed by Kostov (1936) from unbred gene bank accessions of *T. timopheevii* and *T. monococcum*. By contrast, the plant material used in the present study was developed from a selected gene bank accession of *T. timopheevii* and a semi-dwarf breeding line of einkorn, resulting in *T. timococcum* progenies with characteristics different to those previously reported. A previously developed *T. timococcum* (Belea et al. 2005) greatly differed in plant height (124 cm) from that developed in the present study (77 cm), as a tall (143 cm) traditional einkorn was used as male parent instead of a semi-dwarf (66 cm) one. The huge difference between the seed sets of recent and earlier crossings confirms the fact that plant materials can be effectively utilized in the development of synthetic amphiploids only after a strict selection of the possibly most promising parental lines (Belea et al. 2005; Mikó et al. 2013). Through this new combination, valuable einkorn-derived genes for resistance, quality and phenotype were also transferred into the *T. timococcum* genome.

*Triticum timopheevii* somatic chromosomes were earlier identified using the N-banding (Jiang and Gill 1994) and C-banding (Hutchinson and Miller 1982; Badaeva et al. 1993; Rodríguez et al. 2000) techniques. However, fluorescence *in situ* hybridization (FISH) also proved to be an effective tool for obtaining detailed chromosome descriptions of *T. timopheevii*, as shown by Jiang and Gill (1994), Rodríguez et al. (2000), Uhrin et al. (2012) and by the present study. The identification of *T. monococcum* chromosomes using FISH was previously reported by Megyeri et al. (2012). On the basis of these findings, three karyotypes were developed from the three sets of chromosomes originating from *T. timopheevii* (genomes A^t^ and G) and *T. monococcum* (genome A^m^) using the repetitive DNA probes pSc119.2, pTa71 and Afa-family. The present study also proved that the chromosomes of the hybrid, *T. timococcum*, could be easily identified by FISH using the same combination of DNA probes. All the seven A^t^ and seven A^m^ chromosomes could be discriminated from each other. Only chromosomes 2A^m^ and 6A^m^ were difficult to distinguish, but clearer identification could be achieved in the future by the simultaneous application of another repetitive DNA sequence, the SSR probe (GAA)_n_, which gives strong specific telomeric (2A^m^) and centromeric (6A^m^) signals (Megyeri et al. 2012).

No pTa71 signals were found on chromosomes 1A^t^ and 5A^t^, as previously reported by Jiang and Gill (1994), in agreement with the results of Uhrin et al. (2012). McGISH was proved by the present study to be an effective tool for clearly discriminating the G genome from the two A genomes in the hexaploid *T. timococcum*, which also carries species-specific translocations derived from *T. timopheevii* (6A^t^S/1GS and 4GS/4A^t^L).

The phenotypic characteristics of *T. timococcum* were found in the present study to exhibit traits intermediate between the parents. The spikelet shape (intermediate type) was the same as that reported for a previously developed *T. timococcum* by Belea et al. (2005), which also differed in plant height from that developed in the present study, as a tall (143 cm) traditional einkorn was used as male parent instead of a semi-dwarf (66 cm) one. The shorter plant height of the new *T. timococcum* (77 cm) could be advantageous in terms of lodging and drought. Tolerance to drought and the insect vectors of viruses could be enhanced by the inherited pubescence of *T. timococcum*, which could be introduced into wheat together with strong biotic resistance.

Most studies on the utilization of *T. timopheevii* are based on direct crosses with bread wheat (Peusha et al. 1996; Badaeva et al. 1995; Timonova et al. 2012). However, the development of *T. timococcum* will allow crosses to be made at the hexaploid level (bridge-cross), which is thought to be more effective. The task of further research will be to include the best, selected *T. timococcum* lines in bread wheat prebreeding programs using the backcross technique through several generations of *T. aestivum* × *T. timococcum* hybrids, in order to gain useful materials not only for conventional, but also for organic wheat breeders.
